# NGR1 Pretreatment Enhances the Therapeutic Efficacy of Transplanting Cardiomyocytes Derived from Human Induced Pluripotent Stem Cells for Myocardial Infarction

**DOI:** 10.3390/ijms27010475

**Published:** 2026-01-02

**Authors:** Hao Cai, Meng-Ying Huang, Fang-Fang Mou, Qiang-Li Wang, Zhi-Rong Luo, Ping-Ping Lu, Bao-Nian Liu, Liang Hu, Hai-Dong Guo

**Affiliations:** 1 Department of Anatomy, School of Integrative Medicine, Shanghai University of Traditional Chinese Medicine, Shanghai 201203, China; mygame-ch@163.com (H.C.); mengyinghuang2024@163.com (M.-Y.H.); mouff@126.com (F.-F.M.); luozhirong0214@163.com (Z.-R.L.); tikylu@163.com (P.-P.L.); 2 Department of Histoembryology, School of Integrative Medicine, Shanghai University of Traditional Chinese Medicine, Shanghai 201203, China; wlei810@163.com; 3 Academy of Integrative Medicine, Shanghai University of Traditional Chinese Medicine, Shanghai 201203, China; fcchul@zzu.edu.cn

**Keywords:** induced pluripotent stem cell, myocardial infarction, survival, paracrine secretion, heart function

## Abstract

Human induced pluripotent stem cells (hiPSCs) offer significant potential for differentiation and research applications in cardiovascular diseases. When induced differentiated hiPSC-derived cardiomyocytes (hiPSC-CMs) are transplanted into the infarcted myocardial region, they exhibit extremely low survival rates and unsatisfactory therapeutic effects due to ischemia, hypoxia, and immune inflammation in the surrounding environment. To address this issue, we used Panax notoginseng saponin R1 (NGR1), which has demonstrated significant protective effects in prior research, to pretreat hiPSC-CMs before transplantation. Utilizing an in vitro H_2_O_2_ oxidative stress model and a nude mouse myocardial infarction (MI) model, we investigated the mechanism through which NGR1 pretreatment enhances the therapeutic efficacy of hiPSC-CM transplantation. The results revealed that the hiPSC-CMs expressed cTnT. NGR1 did not promote the proliferation of hiPSC-CMs but instead induced elevated levels of p-Akt protein in these cells. Compared to hiPSC-CM transplantation alone, transplantation of hiPSC-CMs pretreated with NGR1 exhibited higher ejection fraction (EF) and fractional shortening (FS) values, along with reduced infarct size and collagen deposition. Additionally, there were more HNA-positive cardiomyocytes in the cardiac tissue, fewer TUNEL-positive signals, and increased VWF-positive and Lyve1-positive signals. Furthermore, the gene expression levels of VEGFC, IGF-1, and SDF-1 were higher. Therefore, NGR1 pretreatment improves the survival of transplanted hiPSC-CMs in tissues, reduces myocardial apoptosis, enhances cardiac function, decreases infarct size and collagen deposition, promotes angiogenesis and lymphangiogenesis, and stimulates paracrine secretion.

## 1. Introduction

Myocardial infarction (MI), as one of the leading causes of mortality worldwide, is a severe disease characterized by myocardial ischemic necrosis, resulting from coronary artery obstruction and severe, persistent ischemia in the myocardial region [1]. Clinical treatments typically involve intravenous thrombolysis, percutaneous coronary intervention (PCI), and other methods aimed at promptly restoring blood supply and minimizing damage. However, issues such as microvascular obstruction and dysfunction, distal embolism, and myocardial ischemia–reperfusion injury (MIRI) following intervention remain unresolved [2]. Furthermore, the irreversible necrosis of myocardial cells after MI leads to alterations in myocardial structure and function, subsequently triggering cardiac remodeling and heart failure.

In recent years, various fields have conducted extensive foundational research on treatments related to MI. Stem cells, including mesenchymal stem cells (MSCs) and human induced pluripotent stem cells (hiPSCs), have garnered significant attention due to their potential for myocardial repair [3]. Among these, hiPSCs stand out because of their ability to proliferate and differentiate in multiple directions, particularly into cardiomyocytes [4]. HiPSCs are sourced from a variety of tissues, which helps avoid ethical concerns and reduces the risk of immune rejection, making them highly valuable for both basic research and clinical applications [5]. When cultured in vitro, hiPSC-CMs typically form a population of rhythmically beating cells but lack the structure of actual cardiac tissue. The harsh environment in transplantation settings, however, limits their survival and functional integration [6]. As a result, improving the survival rate of transplanted hiPSC-CMs and enhancing their integration into cardiac tissue has become a key focus of current research [7].

Traditional pharmacological and interventional approaches struggle to reverse the aforementioned process of myocardial function deterioration after MI [8]. Traditional Chinese medicine and its bioactive compounds have demonstrated significant advances in the prevention and treatment of MI. Notably, NGR1, a principal active component of Panax ginseng, exhibits cardioprotective properties, including anti-inflammatory, antioxidant, and anti-apoptotic effects. Previous studies have indicated that NGR1 can markedly ameliorate MIRI, reduce cardiomyocyte apoptosis, and facilitate the recovery of cardiac function [9]. Its mechanisms involve modulation of multiple signaling pathways, such as activation of the Wnt/β-catenin and JAK2/STAT3 pathways, as well as inhibition of the TAK1-JNK/p38 signaling cascade, thereby regulating cardiomyocyte apoptosis and inflammatory responses [10]. Furthermore, combining NGR1 with other pharmacological agents or nanocarriers has been shown to enhance its cardioprotective efficacy [11]. In our prior work, we observed that NGR1 pretreatment augmented the therapeutic effect of MSC transplantation in MI [12]. In addition, we also found that MSC pretreated with salvianolic acid B improved cardiac function through angiogenesis and paracrine mechanisms after MI [13].

The present study aimed to evaluate whether NGR1 pretreatment could enhance the efficacy of hiPSC-CM transplantation for MI. Specifically, we investigated the effects of NGR1 pretreatment on hiPSC-CM survival and explored the underlying mechanisms, including promoting angiogenesis, lymphangiogenesis and paracrine secretion, with the goal of providing both theoretical and experimental foundations for its potential clinical application.

## 2. Results

### 2.1. Induction of hiPSC Differentiation and Detection of Myocardial Markers

**Figure 1:**
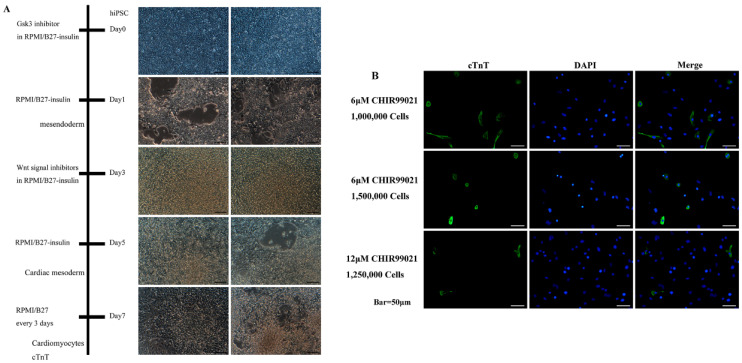
Induction of hiPSC differentiation into cardiomyocytes. (**A**) hiPSCs were induced to undergo differentiation. Scale bar = 100 μm. (**B**) Immunofluorescence staining of cardiac-specific marker cardiac troponin T (cTnT). Scale bar = 50 μm.

Differentiation conditions were optimized by modulating cell density and inhibiting the GSK3 and Wnt signaling pathways. The results indicate that treatment with 6 μM CHIR99021 and seeding at a density of 1 × 10^6^ cells per well efficiently induced differentiation into cardiomyocytes. On the first day of induction, inhibition of signaling pathways led to partial cell death and a reduction in cell density. By the second day, cell density had increased, and by day five, it had stabilized. By day seven, cells exhibited morphology reminiscent of H9c2 cardiomyocytes, and they began spontaneous contractions within seven to ten days, which persisted for approximately one month (Figure 1A). Following differentiation into cardiomyocytes, the cells were transferred to confocal culture dishes and cultured for an additional two days. Immunofluorescence staining revealed robust expression of cTnT, with a substantial proportion of cells exhibiting positive fluorescence under confocal microscopy (Figure 1B).

### 2.2. Transplantation of hiPSC-CMs with NGR1 Pretreatment Improves Cardiac Function

**Figure 2:**
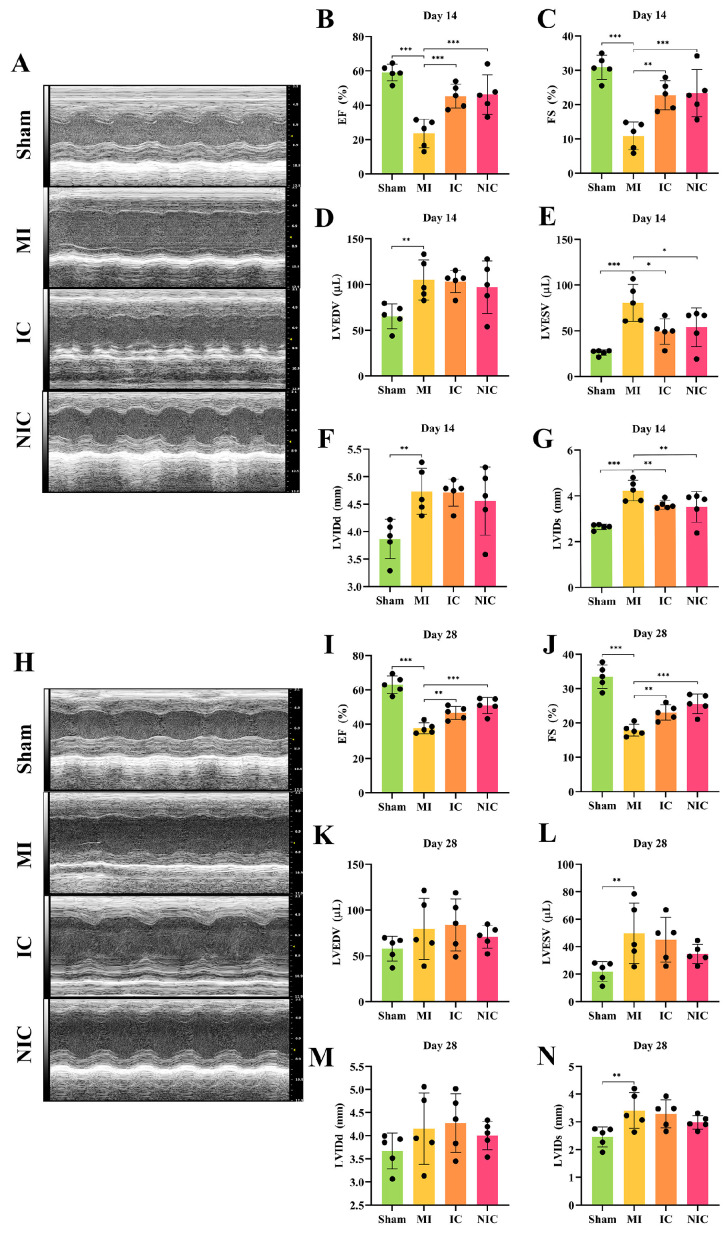
Transplantation of NGR1-pretreated hiPSC-CMs enhanced cardiac function. (**A**) Echocardiography of each group at 2 weeks following acute MI. (**B**–**G**) Statistical analysis of EF, FS, LVEDV, LVESV, LVIDd, and LVIDs at 2 weeks. (**H**) Echocardiography of each group at 4 weeks. (**I**–**N**) Statistical analysis of EF, FS, LVEDV, LVESV, LVIDd, and LVIDs at 4 weeks. *, *p* < 0.05, **, *p* < 0.01, ***, *p* < 0.001. *n* = 5.

Nude mice were administered hiPSC-CMs immediately after inducing MI by ligating the anterior descending branches of the left coronary artery. Echocardiograms taken at 2 and 4 weeks post-transplantation, as shown in Figure 2, revealed significant improvements in various cardiac function parameters. In comparison to the Sham group, the MI group exhibited significant increases in EF, FS, LVESV, and LVIDs (Figure 2B–N). In the IC group, EF and FS were notably improved at 2 weeks compared to the MI group, while LVEDV and LVIDd were higher than both the Sham and MI groups. Furthermore, LVESV and LVIDs were reduced in the IC group relative to the MI group. In the NIC group, both EF and FS were significantly enhanced compared to the MI group, with trends suggesting even better recovery than in the IC group. Additionally, the recovery of LVEDV, LVIDd, LVESV, and LVIDs in the NIC group was superior to that observed in the IC group (Figure 2B–G). LVESV and LVIDs in the IC and NIC groups were significantly lower than those in the MI group, indicating that hiPSC-CM injection effectively alleviated or delayed ventricular remodeling in the early stage of MI. While some compensatory ventricular dilation after MI or transient effects occurred following cell injection, at 4 weeks, cardiac function parameters, including EF and FS in the NIC group, were still significantly improved compared to the MI group, and the trend of recovery was more pronounced than that in the IC group (Figure 2I–N).

### 2.3. NGR1-Pretreated hiPSC-CM Transplantation Reduces Infarct Area and Collagen Deposition

**Figure 3:**
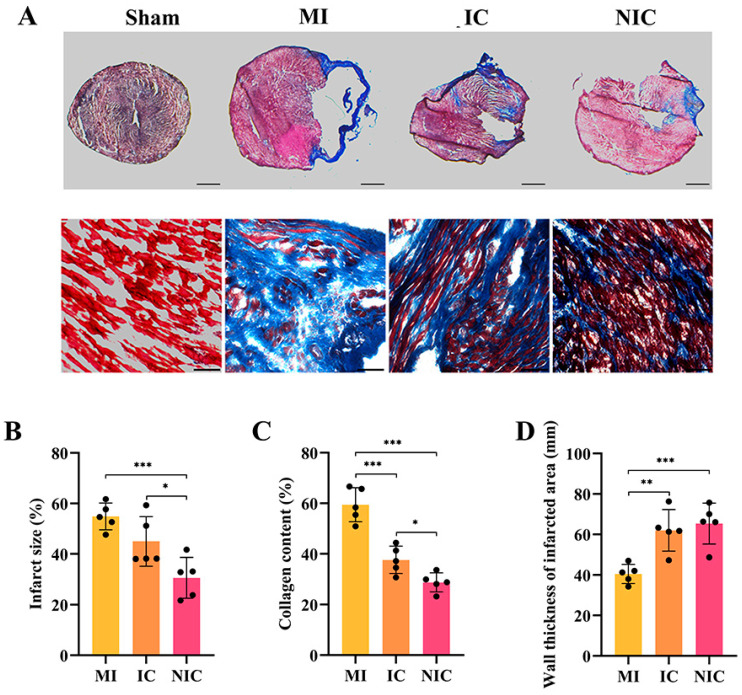
NGR1-pretreated hiPSC-CM transplantation reduced infarct size. (**A**) Masson’s trichrome staining in the infarcted region at 4 weeks. Scale bar (top) = 1 mm. Scale bar (bottom) = 50 μm. (**B**) Statistical analysis of infarct area. (**C**) Statistical analysis of collagen fiber deposition. (**D**) Statistical analysis of ventricular wall thickness in the infarct zone. *, *p* < 0.05, **, *p* < 0.01, ***, *p* < 0.001. *n* = 5.

Masson’s trichrome staining demonstrated that both hiPSC-CM transplantation and NGR1-pretreated hiPSC-CM transplantation reduced the infarct area and collagen deposition. The infarct area and collagen fiber deposition were notably lower in the NGR1-pretreated hiPSC-CM transplantation group compared to the model and IC groups (Figure 3A–C). Additionally, the thickness of the ventricular wall in the infarct zone was significantly reduced in the model group. However, both hiPSC-CM and NGR1-pretreated hiPSC-CM transplantation promoted increases in the ventricular wall thickness (Figure 3D).

### 2.4. NGR1 Pretreatment Enhances Survival of Transplanted hiPSC-CMs

**Figure 4:**
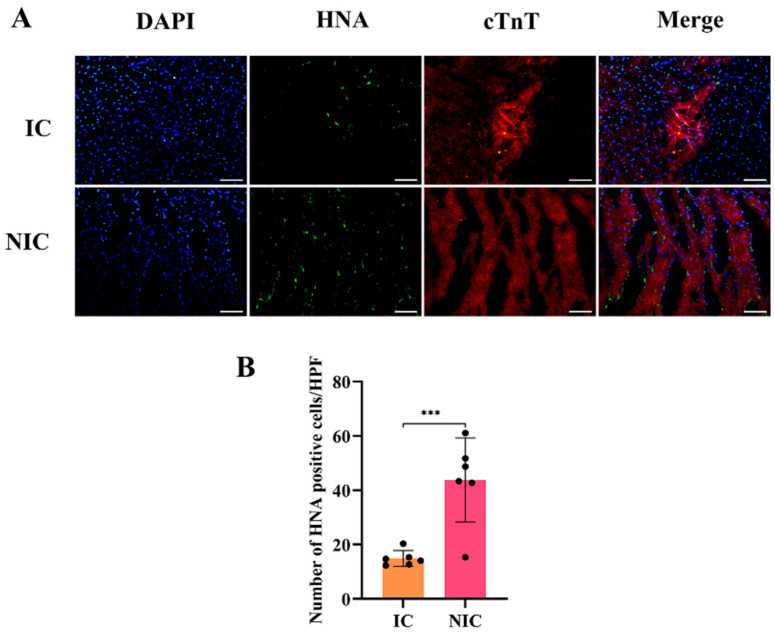
NGR1 pretreatment improved hiPSC-CM survival post-transplantation. (**A**) Immunofluorescence staining of HNA and cTnT was performed on frozen heart sections from nude mice at 4 weeks. (**B**) Statistical analysis of the number of transplanted hiPSC-CMs. ***, *p* < 0.001. Scale bar = 50 μm. *n* = 6.

As the transplanted cells were human-derived, their survival and engraftment were determined through immunofluorescence staining of human nuclear antigen (HNA). HNA and cTnT staining indicated that the survival rate of the transplanted hiPSC-CMs in the NIC group was significantly higher than that in the IC group (Figure 4A,B). The NGR1 pretreatment further increased the survival of the transplanted hiPSC-CMs.

### 2.5. NGR1 Pretreatment Reduces Apoptosis in Cardiac Tissue

**Figure 5:**
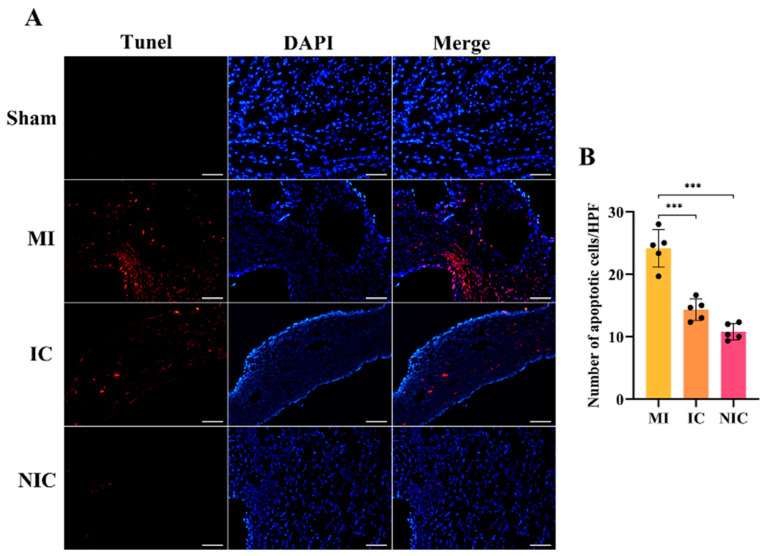
NGR1-pretreated hiPSC-CM transplantation reduced cell apoptosis. (**A**) TUNEL staining of frozen heart sections from nude mice at 4 weeks. (**B**) Number of apoptotic cells in the infarcted regions. ***, *p* < 0.001. Scale bar = 50 μm. *n* = 5.

DAPI and TUNEL staining results revealed a significant increase in apoptotic cells following infarction. Transplantation of hiPSC-CMs reduced the number of apoptotic cells, while the transplantation of NGR1-pretreated hiPSC-CMs led to a more pronounced reduction in apoptosis compared to the hiPSC-CMs-only group (Figure 5A,B).

### 2.6. NGR1-Pretreated hiPSC-CM Transplantation Promotes Angiogenesis

**Figure 6:**
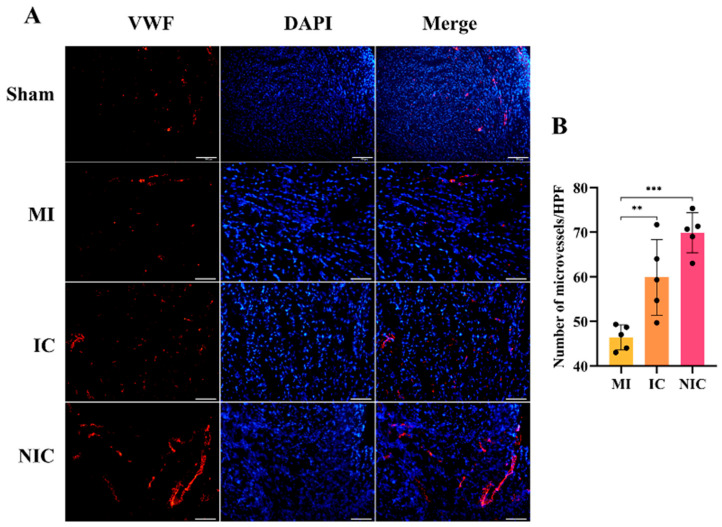
NGR1 pretreatment promoted angiogenesis following hiPSC-CMs transplantation. (**A**) Immunofluorescence staining for vWF in frozen heart sections from nude mice at 4 weeks. (**B**) Statistical analysis of neovascularization in infarcted areas. **, *p* < 0.01, ***, *p* < 0.001. Scale bar = 50 μm. *n* = 5.

von Willebrand Factor (vWF) staining demonstrated that transplantation of hiPSC-CMs significantly increased the number of vWF-positive vessels. The NIC group exhibited a larger number of vWF-positive vessels than the IC group, indicating that transplantation of NGR1-pretreated hiPSC-CMs more effectively promoted angiogenesis in the infarcted region (Figure 6A,B). Angiogenesis can reduce the degree of MI, decrease cardiomyocyte apoptosis and necrosis, improve cardiac remodeling, and also improve the living environment of stem cells.

### 2.7. NGR1-Pretreated hiPSC-CM Transplantation Enhances Lymphangiogenesis

**Figure 7:**
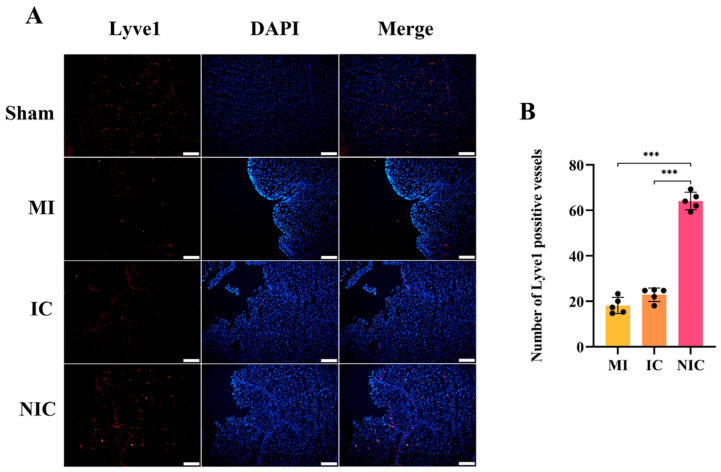
NGR1 pretreatment stimulated lymphangiogenesis following hiPSC-CM transplantation. (**A**) Immunofluorescence staining for LYVE1 at 4 weeks. (**B**) Statistical analysis of lymphatic vessels numbers in infarcted areas. ***, *p* < 0.001. Scale bar = 50 μm. *n* = 5.

Lyve1 staining showed a significant increase in the number of Lyve1-positive vessels following hiPSC-CM transplantation, with the NIC group exhibiting a greater number of Lyve1-positive vessels than the IC group. This suggests that NGR1 pretreatment enhanced the ability of hiPSC-CMs to promote lymphangiogenesis in the infarcted area (Figure 7A,B). Lymphoneogenesis can reduce myocardial edema and interstitial pressure, regulate the immune response, promote the clearance of necrotic tissue, and inhibit cardiac remodeling and fibrosis.

### 2.8. NGR1-Pretreated hiPSC-CM Transplantation Stimulates Paracrine Secretion

**Figure 8:**
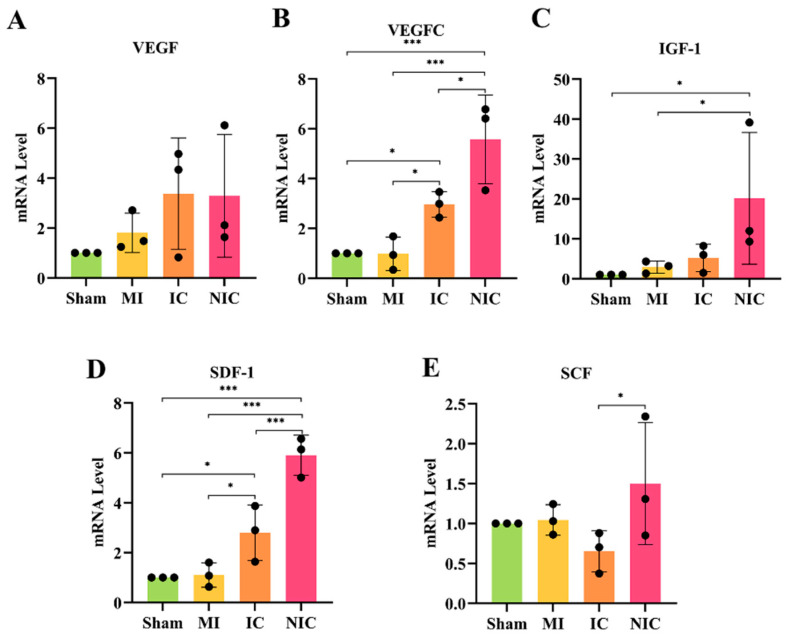
NGR1-pretreated hiPSC-CM transplantation enhanced paracrine secretion. (**A**–**E**) Quantitative RT-PCR analysis was used to assess mRNA expression levels of VEGF, VEGFC, IGF-1, SDF-1, and SCF at 4 weeks. *, *p* < 0.05; ***, *p* < 0.001. *n* = 3.

Quantitative RT-PCR analysis of mRNA levels for SCF, VEGF, IGF-1, SDF-1, and VEGFC in myocardial tissue from the infarcted region of nude mice showed that, compared to the MI group, transplantation of hiPSC-CMs increased the expression of SDF-1. Notably, the levels of VEGFC, IGF-1, and SDF-1 were significantly higher in the NGR1-pretreated hiPSC-CM transplantation group compared to both the hiPSC-CMs-only and MI groups, with statistically significant differences observed (Figure 8A–E).

### 2.9. Regulation of PI3K/Akt Signaling Pathway in hiPSC-CMs by NGR1

To clarify the protective mechanism of NGR1 on hiPSC-CMs, the level of Akt was detected in vitro. Treatment of hiPSC-CMs with 0.1 μM NGR1 resulted in a significant increase in p-Akt levels between 24 and 48 h (Figure S1A). The addition of the PI3K inhibitor LY294002 led to a reduction in p-Akt levels (Figure S1B). However, none of the NGR1 concentrations tested had a significant impact on the proliferation of the hiPSC-CMs (Figure S1C).

## 3. Discussion

Myocardial differentiation of hiPSCs is typically achieved by recapitulating the regulation of key transcription factors and signaling pathways involved in embryonic heart development [14]. Notably, transcription factors such as GATA4 and NKX2.5 are tightly regulated, and the temporal activation and inhibition of the Wnt/β-catenin signaling pathway constitutes a critical determinant for efficient myocardial regeneration [15]. Direct transplantation of allogeneic hiPSCs, however, may elicit severe immune rejection. Gene-editing strategies, including B2M knockout and HLA-E overexpression, can generate hiPSCs and their derivatives with reduced immunogenicity, thereby enhancing transplant survival [16]. In vitro-directed differentiation of hiPSCs can produce substantial numbers of hiPSC-CMs exhibiting characteristic myocardial markers and contractile function [17]. Preclinical studies have demonstrated that transplanted hiPSC-CMs can integrate into host myocardial tissue, improve cardiac contractility, and mitigate myocardial fibrosis, although these effects are often limited [18]. Beyond immunogenicity, the ischemic, hypoxic, and immunologically stressful environment at the transplantation site contributes to significant loss of transplanted cells. To enhance the therapeutic efficacy of hiPSC and hiPSC-CM transplantation, combination strategies have emerged as a key research focus. Incorporation of collagen-based scaffolds to simulate the myocardial extracellular matrix can facilitate the colonization and functional integration of hiPSC-CMs [19]. Gene-edited hiPSC-CMs can further improve transplanted cell survival and myocardial repair by promoting angiogenesis and ameliorating hypoxic conditions [20]. Additionally, pharmacological co-treatment with stem cell transplantation has shown promise in multiple preclinical studies. Our previous work demonstrated that NGR1 combined with MSC transplantation significantly enhances both the survival and therapeutic efficacy of transplanted cells [12,13].

As a diterpenoid saponin primarily derived from Panax notoginseng, NGR1 exhibits anti-inflammatory and antioxidant properties, making it a promising candidate for combinatory therapeutic applications [21]. Our preliminary investigations demonstrated that NGR1 enhances the therapeutic efficacy of MSC transplantation in MI through activation of the PI3K/Akt signaling pathway. This pathway functions as a critical regulatory hub for cell survival, proliferation, immune response, and metabolism, modulating cellular responses to extracellular stimuli and intracellular environmental changes [22]. Phosphatidylinositol 3-kinase (PI3K) is a key signal transduction molecule that is activated by receptor tyrosine kinases (RTKs) and other membrane receptors. Upon activation, PI3K catalyzes the conversion of membrane phosphatidylinositol 4,5-bisphosphate (PIP2) into phosphatidylinositol 3,4,5-triphosphate (PIP3). The production of PIP3 recruits protein kinase B (Akt) to the cell membrane, promoting its phosphorylation and conformational activation [23]. Activated Akt phosphorylates downstream targets such as forkhead box protein O1 (FoxO1), thereby inhibiting pro-apoptotic proteins, enhancing cell survival, reducing the secretion of pro-inflammatory cytokines (e.g., TNF-α and IL-6), and regulating glucose metabolism and protein synthesis [24]. Activation of the PI3K/Akt pathway also upregulates intracellular antioxidant enzymes, including superoxide dismutase and catalase, and mitigates inflammation by cross-regulating pathways such as NF-κB and MAPK, ultimately suppressing oxidative stress- and inflammation-induced apoptosis [25]. Our data indicate that NGR1 pretreatment did not significantly enhance the proliferation of hiPSC-CMs but markedly increased Akt phosphorylation. The PI3K inhibitor LY294002 effectively suppressed this phosphorylation. Compared to direct hiPSC-CM transplantation, NGR1-pretreated hiPSC-CMs exhibited improved survival in cardiac tissue, reduced myocardial apoptosis, enhanced cardiac function, and decreased infarct size and collagen deposition. These findings possibly suggest that NGR1 pretreatment promotes hiPSC-CM survival post-transplantation and improves the therapeutic efficacy of hiPSC-CM transplantation in MI via PI3K/Akt pathway activation.

The cardiac microvascular system, comprising capillaries, arterioles, and venules, is essential for myocardial perfusion, and its reconstruction is critical for tissue survival and functional recovery [26]. Physiologically, microvessels regulate blood flow, ensuring adequate oxygen and nutrient delivery to the myocardium [27]. Following MI, angiogenesis—mediated by endothelial cell proliferation, migration, and lumen formation—is crucial for tissue repair and is regulated by diverse growth factors [28]. This study found that NGR1 pretreatment promoted hiPSC-CM angiogenesis and increased the expression of related factors.

Cardiac lymphatic vessels, distributed throughout the myocardium and epicardium, are vital for fluid clearance and modulation of inflammatory responses [29]. Promoting lymphangiogenesis accelerates the removal of inflammatory cells, mitigates myocardial fibrosis, inhibits pathological remodeling, and improves cardiac function [30]. Lymphatic endothelial cells, which express the marker Lyve1, are essential for lymphatic vessel formation and functional maintenance [31]. The VEGFC/VEGFR-3 axis is a central signaling pathway that regulates cardiac lymphangiogenesis, facilitating lymphatic neogenesis and protecting cardiac function by modulating intracellular signaling and reducing cardiomyocyte apoptosis and inflammation [32]. We observed that transplantation of NGR1-pretreated hiPSC-CMs significantly increased VEGFC expression and the density of Lyve1-positive lymphatic vessels, indicating that this strategy promotes lymphangiogenesis and improves cardiac function via VEGFC regulation.

Angiogenesis and lymphangiogenesis are interconnected processes in cardiac repair, sharing overlapping molecular mechanisms. Following myocardial injury, microvascular growth coincides with chronic inflammation and fibrosis, whereas lymphatic dysfunction exacerbates tissue edema and inflammation [33]. Endothelial injury and endothelial-to-mesenchymal transition reduce vascular density and function, while TGF-β secretion activates cardiac fibroblasts [34]. Lymphatic impairment hinders the clearance of pro-inflammatory factors and necrotic cells, perpetuating inflammation and extracellular matrix accumulation [35]. Therefore, coordinated regulation of vascular and lymphatic networks is essential for effective myocardial repair. VEGFC, through VEGFR-3 activation, promotes lymphangiogenesis and also contributes to angiogenesis via VEGFR-2, highlighting the dual vascular and lymphatic remodeling facilitated by NGR1 in myocardial tissue repair [36].

SDF-1 is a chemokine that directs stem cell recruitment, migration, and engraftment in ischemic regions via chemotactic gradients [37,38]. SDF-1 mediates the PI3K/Akt pathway, reducing apoptosis while promoting cell migration and differentiation [39]. Additionally, SDF-1 stimulates the secretion of reparative factors, including IGF-1 and SCF, to enhance myocardial repair [40]. IGF-1 inhibits cardiomyocyte apoptosis through regulation of anti-apoptotic proteins such as Bcl-2, promotes angiogenesis and lymphangiogenesis via VEGFR-3, activates the PI3K/Akt/mTOR axis, and stimulates lymphatic endothelial progenitor proliferation, thereby improving cardiac function [41,42]. IGF-1 also interacts with NF-κB signaling to reduce inflammation and attenuate ischemic injury [43]. In our study, transplantation of NGR1-pretreated hiPSC-CMs significantly elevated myocardial expression of VEGFC, SDF-1, and IGF-1, collectively promoting myocardial repair through anti-apoptotic, antioxidant, and pro-regenerative mechanisms. However, the specific mechanism by which NGR1 pretreatment promotes the effects of hiPSC-CM against myocardial injury still needs further study.

Our research demonstrates that the transplantation of hiPSC-CMs pre-treated with NGR1 effectively mitigates MI-induced ventricular remodeling, preserves cardiac function, and enhances the resistance of the transplanted stem cells to oxidative stress-induced apoptosis. NGR1 pretreatment is a simple and feasible method with good clinical translation prospects. Although this study is in its preliminary stages, focusing on animal and cellular models, it provides valuable insights for the development of drug–stem cell combination therapies with potential clinical translational relevance. It is also necessary to improve the maturity of hiPSC-CMs, avoid the risk of arrhythmia, and optimize the dosage of NGR1. We aim to further explore and refine this system by improving the resistance of transplanted stem cells to oxidative stress, immune modulation, and other challenges [44]. This will help address electrophysiological issues, such as the integration of myocardial tissue post-transplantation [45]. Additionally, it is crucial to optimize the drug dosing and stem cell differentiation protocols for various transplantation contexts, conduct safety assessments during the translational process, and refine the overall methodology to ensure the biological integrity of the product, compliance with current Good Manufacturing Practices (cGMP), and patient safety [46]. We believe that emerging omics technologies and interdisciplinary materials have the potential to accelerate progress in the exploration and treatment of cardiovascular diseases [47,48].

## 4. Materials and Methods

### 4.1. hiPSC Cultivation

Frozen cells (National Collection of Authenticated Cell Cultures, Shanghai, China) were quickly thawed in a 37 °C water bath and transferred to 5 mL of mTeSR1 culture medium (STEMCELL Technologies, 85857, Vancouver, BC, Canada). The cells were centrifuged at 200× *g* for 5 min to remove the supernatant. Then, the cells were resuspended in 6 mL of mTeSR1 culture medium containing 5 mM Y27632 (Tocris, 1254, Bristol, UK) and transferred to a 6-well plate with matrix gel. After 24 h, the cell culture medium was replaced with mTeSR1 culture medium without Y27632. The culture medium was changed daily. When the cells reached 80–90% confluence, they were digested in a 37 °C incubator with Versene (Thermo Fisher, 15040066, Waltham, MA, USA) for 4 min. Digestion was stopped with 3 mL of mTeSR1 culture medium containing 5 mM Y27632. After centrifugation and resuspension, the cells were cultured in 9 mL of mTeSR1 culture medium containing 5 mM Y27632. The ratio was 1:6.

### 4.2. Induced Differentiation of hiPSC

The cells in the 6-well plate were digested with 1 mL Accutase (Thermo Fisher, 00-4555-56) for 8 min, centrifuged at 200× *g* for 5 min, resuspended in mTeSR1 culture medium and counted. The cells were then resuspended in Y27632 and mTeSR1 culture medium at a concentration of 2 million/mL. Then, 0.5, 0.75, 1 million, 1.25 million, and 1.5 million cells were added to each well of a 12-well plate, and the culture medium was supplemented to 1 mL with Y27632 and mTeSR1. The time point was −4 days. During the following 3 days, the culture medium was replaced every day. On day 0, each well was replaced with 2 mL of RPMI (Thermo Fisher, 11875093)/B27 (Thermo Fisher, 17504044) insulin culture medium containing 12 μM CHIR99021 (Selleckchem, S1263, Houston, TX, USA). On the first day, it was switched to RPMI/B27 insulin culture medium. On the third day, it was replaced with 2 mL of combined medium, which consisted of 1 mL of culture medium, 1 mL of RPMI/B27 insulin culture medium, and 5 μM IWP2 (Merck, I0536, Darmstadt, Germany). Then, it was replaced with 2 mL of RPMI/B27 insulin culture medium at 5 days. The culture medium was replaced with RPMI/B27 every 7 days and every 3 days thereafter. The above scheme is based on verified relevant papers [49].

### 4.3. Detection of Cardiac Cell Markers in hiPSC-CM

The cells in each well were washed with PBS, digested with 0.5 mL of 0.25% Trypsin EDTA (Corning Cellgro 25-051-CI, Corning, NY, USA) at 37 °C for 5 min. Then, 2 mL of RPMI20 culture medium was added. The cells were centrifuged at 200× *g* for 5 min and resuspended in Y27632 and mTeSR1 culture medium at a concentration of 100,000/mL. Subsequently, 1 mL of 0.1% gelatin was added to each well. Two days later, the cells were fixed with 4% formaldehyde at room temperature for 15 min, followed by two washes with PBS. They were then permeabilized with 0.4% Tritonx-100 for 5 min. Subsequently, the cells were incubated at room temperature for 1 h with anti-cardiac troponin T (cTnT) primary antibody (Abcam, ab209813, Cambridge, UK), washed three times with PBS, and incubated in the dark for 30 min with secondary antibody. After three additional PBS washes, the cells were photographed.

### 4.4. CCK8 Testing

A total of 10,000 cells were seeded into each well of a 96-well plate and cultured in RPMI/B27 culture medium containing the corresponding concentration of NGR1. After 24 h, the culture medium was discarded from each well and replaced with RPMI/B27 culture medium containing 10% CCK8. The cells were incubated in the incubator for 1 h and the absorbance in each well was measured at 450 nm. The concentration and duration of all NGR1-related treatments in this study were based on our previous exploration [12].

### 4.5. Western Blot

After treatment with corresponding concentrations of LY294002 (Selleck.cn, S1105, Shanghai, China) and NGR1, proteins were digested and extracted using RIPA (Beyotine, P0013B, Shanghai, China. The concentration was balanced using the BCA method. LDS was added and denatured at 70 °C for 10 min, followed by 200 V electrophoresis for 25 min and 100 V membrane transfer for 90 min. The primary antibodies p-Akt (Ser473) (CST, 4060, Danvers, MA, USA), Akt (C67E7) (CST, 4691), and GAPDH (14C10) (CST, 2118) were incubated at 1:1000 at 4 °C overnight, and the secondary antibodies were incubated at room temperature for 1 h before exposure and photography.

### 4.6. Nude Mouse Model and Cell Transplantation

Adult nude mice weighing 19–21 g were divided into a sham surgery group (Sham, injected an equal volume of physiological saline solution) with 8 mice, a model group (MI) with 12 mice, an hiPSC-CM injection group (IC) with 12 mice, and an NGR1-pretreated hiPSC-CM injection group (NIC) with 12 mice, with NGR1 pretreatment for 24 h. The MI, IC, and NIC groups of nude mice were anesthetized with isoflurane and fixed. A 1cm incision was made near the third and fourth intercostal spaces on the left side of the sternum, and the skin was bluntly separated from the intercostal spaces to squeeze out the heart. The left anterior descending branch (LAD) of the coronary artery was ligated 1mm below the left atrial appendage. After ligation, 500,000 hiPSC-CM or NGR1-pretreated hiPSC-CM were injected into the left ventricular infarction zone at 3 sites of nude mice in the IC and NIC groups. The hearts of the nude mice were returned to the chest cavity, and the skin was sutured. The mortality rate was about 40%, with a total of 11 deaths, including 4 in the MI group, 4 in the IC group, and 3 in the NIC group. The mice were humanely euthanized by an overdose of carbon dioxide inhalation.

### 4.7. Echocardiography

At 2 and 4 weeks of modeling, the nude mice were anesthetized with isoflurane and fixed. A probe was placed on the left chest wall, and a 30 MHz probe was used to obtain left ventricular M-echocardiography. Then, ejection fraction (EF), left ventricular short axis fractional shortening (FS), left ventricular end diastolic volume (LVEDV), left ventricular end systolic volume (LVESV), left ventricular diastolic diameter (LVIDd), and left ventricular systolic diameter (LVIDs) were measured and calculated.

### 4.8. Masson’s Trichrome Staining

After the heart was fixed and dehydrated, it was embedded and frozen with OCT, and then sliced. The frozen slices were dried at room temperature for 10 min, washed three times with PBS, and stained overnight with Bouin at room temperature. Subsequently, Masson’s trichrome staining was performed according to the manufacturer’s instructions. For morphometric parameters, the percentage of infarct area was obtained by dividing the blue collagen area by the left ventricular area in the 8 bit mode of ImageJ (2.1.0).

### 4.9. Immunofluorescence Staining

The frozen sections were dried at room temperature for 10 min, washed three times with PBS, and the 0.5% TritonX-100 membrane was broken at room temperature for 15 min. Sections were blocked with goat serum at 37 °C for 30 min and then incubated with primary antibody overnight at 4 °C and washed three times with PBS. Subsequently, the sections were incubated with secondary antibody at room temperature in the dark for 1 h. After being washed with PBS again, the cell nucleus was co-stained with DAPI in the dark for 10 min. After sealing, the slices were photographed.

### 4.10. RT-qPCR Detection

Total RNA was extracted from ventricular samples, and the concentration was measured. cDNA was obtained through reverse transcription using the Adamas life 5 × Reverse Transcription Mix kit (Adamas life, G8041-0010, Shanghai, China). A 20 μL reverse transcription system was prepared at 42 °C for 15 min and 95 °C for 30 s. The cDNA was diluted 5 times. An RT-qPCR system of 20 μL was prepared with qPCR SYBR Master Mix. The reaction procedure involved pre-denaturation at 95 °C for 15 min, followed by fluorescence collection at 60 °C for 30 s after 10 s at 95 °C for 40 cycles of denaturation. The dissolution curve was adapted using a machine program. The primer sequence of vascular endothelial growth factor A (VEGF), vascular endothelial growth factor C (VEGFC), insulin-like growth factor-1 (IGF-1), stromal cell-derived factor-1 (SDF-1), stem cell factor (SCF), and glyceraldehyde-3-phosphate dehydrogenase (GAPDH) are shown in Table 1.

### 4.11. Statistical Analysis

All statistical analyses were performed using IBM SPSS 27. Continuous variables were expressed as mean ± standard deviation (SD) for normally distributed data or median (interquartile range) for non-normally distributed data. The normality of data distribution was assessed using the Shapiro–Wilk test. Group comparisons for continuous variables were conducted using Student’s *t*-test (for normally distributed data) or the Mann–Whitney U test (for non-normally distributed data). For comparisons among more than two groups, one-way ANOVA was performed, followed by Bonferroni’s post hoc test for multiple comparisons. Correlation analysis was performed using Pearson’s correlation coefficient for normally distributed data or Spearman’s rank correlation coefficient for non-normally distributed data. Univariate and multivariate Cox proportional hazards models were employed to estimate hazard ratios (HRs) and their 95% confidence intervals (CIs). A two-sided *p*-value < 0.05 was considered statistically significant. The sample size was determined based on a power analysis with an α of 0.05 and β of 0.20 (power of 80%).

### 4.12. Experimental Flowchart

**Figure 9:**
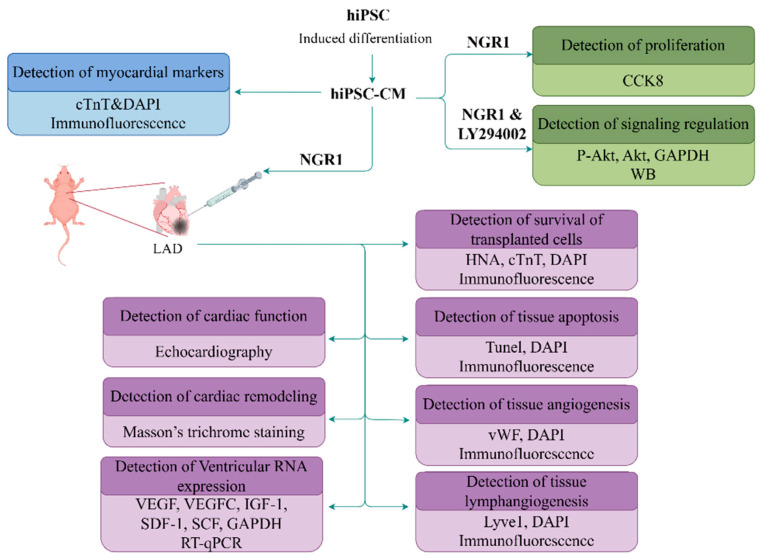
A complete protocol figure for each type of experiment. The blue section represents the detection of the differentiation results derived from hiPSC. The green section represents the detection of the activity and protein expression of hiPSC-CM after treatment with NGR1 and related pathway inhibitors. The purple section represents the detection of the relevant effects on the cardiovascular system of nude mice after hiPSC-CM transplanting.

To facilitate a quick understanding of the design of this study, all the schemes presented in this study can be seen in the figure below (Figure 9).

## 5. Conclusions

hiPSC can be efficiently differentiated into cardiomyocytes through modulation of GSK3 and Wnt signaling and optimization of cell density. NGR1 enhances p-Akt levels in hiPSC-CMs and, when used as a pretreatment, improves the therapeutic efficacy of hiPSC-CM transplantation in MI by promoting graft survival and activating paracrine repair mechanisms.

## Figures and Tables

**Table 1:** Primer sequences.

Gene	Forward Primer	Reverse Primer
VEGF	CTGCCGTCCGATTGAGACC	CCCCTCCTTGTACCACTGTC
VEGFC	GAGGTCAAGGCTTTTGAAGGC	CTGTCCTGGTATTGAGGGTGG
IGF-1	CTGGACCAGAGACCCTTTGC	GGACGGGGACTTCTGAGTCTT
SDF-1	GCAGCCTTTCTCTTCTTCTGTC	ACTCCAAACTGTGCCCTTCA
SCF	GAATCTCCGAAGAGGCCAGAA	GCTGCAACAGGGGGTAACAT
GAPDH	AGGCCGGTGCTGAGTATGTC	TGCCTGCTTCACCACCTTCT

## Data Availability

The original contributions presented in this study are included in the article/Supplementary Materials. Further inquiries can be directed to the corresponding authors.

## Supplementary Materials

The following supporting information can be downloaded at: https://www.mdpi.com/article/10.3390/ijms27010475/s1.

## Abbreviations

NGR1	Panax notoginseng saponin R1
hiPSC	human induced pluripotent stem cell
hiPSC-CMs	human induced pluripotent stem cell derived cardiomyocytes
LAD	left anterior descending branch
MI	myocardial infarction
EF	ejection fraction
FS	fractional shortening
LVEDV	left ventricular end diastolic volume
LVESV	left ventricular end systolic volume
LVIDd	left ventricular diastolic diameter
LVIDs	left ventricular systolic diameter
MSCs	mesenchymal stem cells
VEGF	vascular endothelial growth factor A
VEGFC	vascular endothelial growth factor C
IGF-1	insulin-like growth factor-1
SDF-1	stromal cell-derived factor-1
SCF	stem cell factor
GAPDH	glyceraldehyde-3-phosphate dehydrogenase
MIRI	myocardial ischemia-reperfusion injury
HNA	human nuclear antigen
cTnT	cardiac troponin T
vWF	von Willebrand Factor
PCI	percutaneous coronary intervention
PI3K	phosphatidylinositol 3-kinase
RTKs	receptor tyrosine kinases
PIP2	phosphatidylinositol 4,5-bisphosphate
PIP3	phosphatidylinositol 3,4,5-triphosphate
Akt	protein kinase B
FoxO1	Forkhead box protein O1
Wnt	Wingless-related integration site
JAK2	Janus Kinase 2
STAT3	Signal Transducer and Activator of Transcription 3
TAK1	TGF-β-activated kinase 1
JNK	c-Jun N-terminal kinase
p38	p38 Mitogen-Activated Protein Kinase
