# Supplementary material for: NGR1 Pretreatment Enhances the Therapeutic Efficacy of Transplanting Cardiomyocytes Derived from Human Induced Pluripotent Stem Cells for Myocardial Infarction
Source: Int J Mol Sci. 2026 Jan 2;27(1):475. doi: 10.3390/ijms27010475 (PMC12786578; doi:10.3390/ijms27010475)
Supplement: Supplementary file 1 [file ijms-27-00475-s001.zip › ijms-4031527-supplementary.pdf]

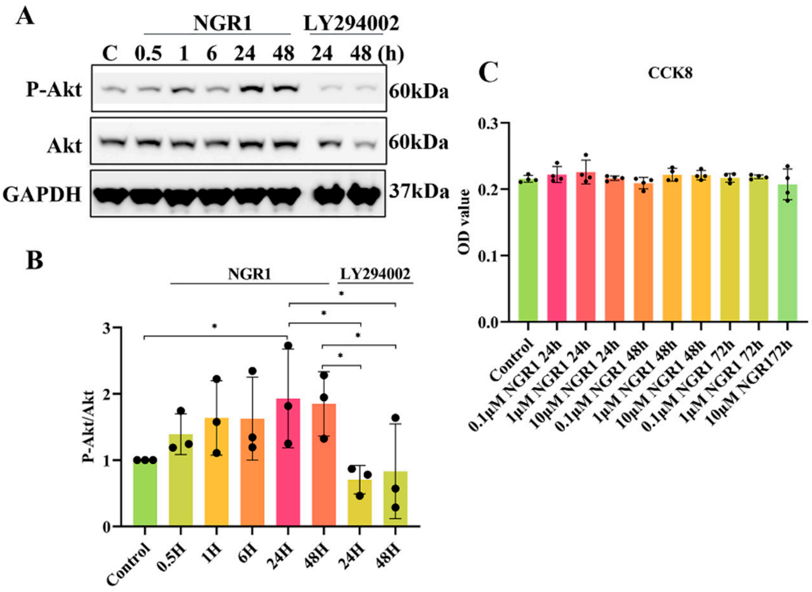

Figure S1. Regulation of PI3K/Akt signaling in hiPSC-CMs by NGR1.

A. Following treatment with 0.1 μmol/L NGR1 and the addition of LY294002, changes of p-Akt levels in hiPSC-CMs were detected by WB at various time points (NGR1 at 0.5h, 1h, 6h, 24h, 48h and LY294002 with NGR1 at 24h, 48h). B. The p-Akt/Akt ratio was also assessed at different time intervals. n=3. C. Proliferation of hiPSC-CMs was evaluated using the CCK-8 assay following treatment with varying concentrations of NGR1 for 24, 48, and 72 hours. The number of cells per well was 100000. n=4. *, $P < 0.05$.
